# 4-[(6-Chloro-2-pyrid­yl)meth­oxy]-3-(2,4-dichloro­phen­yl)-1-oxaspiro­[4.5]dec-3-en-2-one

**DOI:** 10.1107/S1600536809010101

**Published:** 2009-03-25

**Authors:** Liang-zhong Xu, Jin Huang, Qun-qun Su, Wei Guo

**Affiliations:** a College of Chemistry and Molecular Engineering, Qingdao University of Science and Technology, Qingdao 266042, People’s Republic of China

## Abstract

In the title compound, C_21_H_18_Cl_3_NO_3_, the cyclo­hexane ring is in a chair conformation. The five-membered ring forms a dihedral angle of 69.89 (2)° with the benzene ring. The dihedral angle between the benzene and pyridine rings is 14.03 (7)°.

## Related literature

For the biological activity of the title compound and a similar structure, see: Thomas *et al.* (2003 ▶). For its preparation, see: Lu *et al.* (2008 ▶); Sarcevic *et al.* (1973 ▶). For puckering parameters, see: Cremer & Pople (1975 ▶).
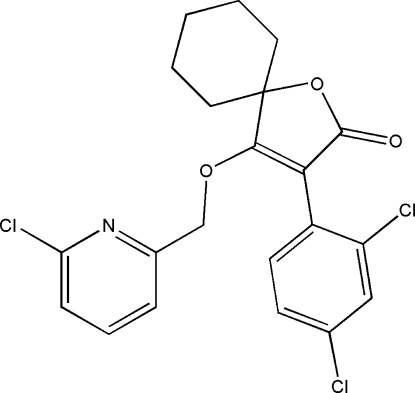


## Experimental

### 

#### Crystal data

C_21_H_18_Cl_3_NO_3_
*M* *_r_* = 438.71
Monoclinic, 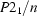

*a* = 7.2457 (14) Å
*b* = 13.108 (3) Å
*c* = 21.054 (4) Å
β = 95.77 (3)°
*V* = 1989.5 (7) Å^3^
*Z* = 4
Mo *K*α radiation
μ = 0.48 mm^−1^
*T* = 113 K
0.18 × 0.12 × 0.06 mm

#### Data collection

Rigaku Saturn diffractometer
Absorption correction: multi-scan (*CrystalClear*; Rigaku, 2005 ▶) *T*_min_ = 0.918, *T*_max_ = 0.972
14740 measured reflections
3519 independent reflections
3191 reflections with *I* > 2σ(*I*)
*R*_int_ = 0.041

#### Refinement

*R*[*F* ^2^ > 2σ(*F* ^2^)] = 0.034
*wR*(*F* ^2^) = 0.089
*S* = 1.08
3519 reflections
253 parameters
H-atom parameters constrained
Δρ_max_ = 0.18 e Å^−3^
Δρ_min_ = −0.31 e Å^−3^

### 

Data collection: *CrystalClear* (Rigaku, 2005 ▶); cell refinement: *CrystalClear*; data reduction: *CrystalClear*; program(s) used to solve structure: *SHELXTL* (Sheldrick, 2008 ▶); program(s) used to refine structure: *SHELXTL*; molecular graphics: *SHELXTL*; software used to prepare material for publication: *SHELXTL*.

## Supplementary Material

Crystal structure: contains datablocks I, global. DOI: 10.1107/S1600536809010101/bx2196sup1.cif

Structure factors: contains datablocks I. DOI: 10.1107/S1600536809010101/bx2196Isup2.hkl

Additional supplementary materials:  crystallographic information; 3D view; checkCIF report

## Figures and Tables

**Fig. 1.**
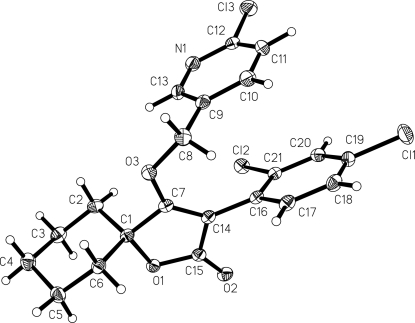
View of the title compound (I), with displacement ellipsoids drawn at the 40% probability level.

## supplementary crystallographic information

### Comment

The goal of the  synthesis of the  title compound (I) is to obtain compounds with biological activity (Thomas *et al.*, 2003). We report here the crystal structure of (I), Fig.1.The cyclohexane ring is a chair conformation [Puckering Amplitude (Q_T_) = 0.5531 (18) Å, θ = 4.39 (19) °, φ = 142 (3) °] (Cremer & Pople, 1975).The five membered ring form a dihedral angle of 69.89 (2)° with the benzene ring. In the crystal structure, the molecular packing is stabilized by one intermolecular C—H···O hydrogen bond.

### Experimental

3-(2,4-Dichlorophenyl)-2,4-dioxo-1-oxaspiro[4.5]decane (6.26 g ;20.0 mmol), was suspended in a solution of sodium carbonate (1.08 g ;10.2 mmol) in 40 ml of water in a flask equipped with stirrer, water separator and reflux condenser. Toluene (80 ml) was added after 0.5 h, the mixture was heated to dehydration. Then 2-chloro-6-(chloromethyl)pyridine 3.56 g (22.0 mmol) and *N*,*N*-dimethylformamide(DMF)  (40 ml) were added while maintaining the temperature at 100° C for 4 h. Upon cooling at room temperature. Then water (40 ml) was added. The mixture was extracted with CH_2_Cl_2_ (35 ml) and the organic layer was washed with water and dried over sodium sulfate. The excess CH_2_Cl_2_ was removed on a water vacuum pump to obtain the oil product which was crystallized from methanol to afford the title compound 7.89 g (90% yield) (Lu *et al.*, 2008; Sarcevic *et al.*, 1973). Single crystals suitable for X-ray measurement were obtained by recrystallization from the mixture of acetone and methanol at room temperature.

### Refinement

All C-bound H atoms were placed in calculated positions, with C—H = 0.93 or 0.97 Å, and included in the final cycles of refinement using a riding model, with *U*_iso_(H) = 1.2*U*_eq_(C) for the aryl and methylene H atoms.

### Crystal data

**Table:**

C_21_H_18_Cl_3_NO_3_	*F*(000) = 904
*M**_r_* = 438.71	*D*_x_ = 1.465 Mg m^−^^3^
Monoclinic, *P*2_1_/*n*	Mo *K*α radiation, λ = 0.71073 Å
Hall symbol: -P 2yn	Cell parameters from 5265 reflections
*a* = 7.2457 (14) Å	θ = 1.6–28.0°
*b* = 13.108 (3) Å	µ = 0.48 mm^−^^1^
*c* = 21.054 (4) Å	*T* = 113 K
β = 95.77 (3)°	Platelet, colourless
*V* = 1989.5 (7) Å^3^	0.18 × 0.12 × 0.06 mm
*Z* = 4	

### Data collection

**Table:**

Rigaku Saturn diffractometer	3519 independent reflections
Radiation source: rotating anode	3191 reflections with *I* > 2σ(*I*)
confocal	*R*_int_ = 0.041
ω scans	θ_max_ = 25.0°, θ_min_ = 1.8°
Absorption correction: multi-scan (*CrystalClear*; Rigaku, 2005)	*h* = −8→8
*T*_min_ = 0.918, *T*_max_ = 0.972	*k* = −15→15
14740 measured reflections	*l* = −25→25

### Refinement

**Table:**

Refinement on *F*^2^	Primary atom site location: structure-invariant direct methods
Least-squares matrix: full	Secondary atom site location: difference Fourier map
*R*[*F*^2^ > 2σ(*F*^2^)] = 0.034	Hydrogen site location: inferred from neighbouring sites
*wR*(*F*^2^) = 0.089	H-atom parameters constrained
*S* = 1.08	*w* = 1/[σ^2^(*F*_o_^2^) + (0.053*P*)^2^] where *P* = (*F*_o_^2^ + 2*F*_c_^2^)/3
3519 reflections	(Δ/σ)_max_ = 0.001
253 parameters	Δρ_max_ = 0.18 e Å^−^^3^
0 restraints	Δρ_min_ = −0.31 e Å^−^^3^

### Special details

**Table:**

Geometry. All e.s.d.'s (except the e.s.d. in the dihedral angle between two l.s. planes) are estimated using the full covariance matrix. The cell e.s.d.'s are taken into account individually in the estimation of e.s.d.'s in distances, angles and torsion angles; correlations between e.s.d.'s in cell parameters are only used when they are defined by crystal symmetry. An approximate (isotropic) treatment of cell e.s.d.'s is used for estimating e.s.d.'s involving l.s. planes.
Refinement. Refinement of *F*^2^ against ALL reflections. The weighted *R*-factor *wR* and goodness of fit *S* are based on *F*^2^, conventional *R*-factors *R* are based on *F*, with *F* set to zero for negative *F*^2^. The threshold expression of *F*^2^ > σ(*F*^2^) is used only for calculating *R*-factors(gt) *etc*. and is not relevant to the choice of reflections for refinement. *R*-factors based on *F*^2^ are statistically about twice as large as those based on *F*, and *R*- factors based on ALL data will be even larger.

### Fractional atomic coordinates and isotropic or equivalent isotropic displacement parameters (Å^2^)

**Table:**

	*x*	*y*	*z*	*U*_iso_*/*U*_eq_	
Cl1	−0.28480 (7)	−0.08999 (4)	0.06950 (2)	0.03906 (15)	
Cl2	−0.06561 (6)	0.21586 (3)	0.228775 (19)	0.02533 (13)	
Cl3	0.36534 (7)	0.22352 (4)	0.06907 (2)	0.03617 (15)	
O1	0.00146 (14)	0.14206 (8)	0.42592 (5)	0.0204 (3)	
O2	−0.26135 (15)	0.13568 (9)	0.35960 (5)	0.0248 (3)	
O3	0.35068 (14)	0.00872 (8)	0.35405 (5)	0.0226 (3)	
N1	0.4102 (2)	0.18419 (11)	0.19104 (7)	0.0264 (3)	
C1	0.1925 (2)	0.10733 (12)	0.42562 (7)	0.0188 (3)	
C2	0.3193 (2)	0.20111 (12)	0.42615 (8)	0.0239 (4)	
H2A	0.4436	0.1795	0.4190	0.029*	
H2B	0.2745	0.2464	0.3915	0.029*	
C3	0.3264 (2)	0.25889 (13)	0.48925 (8)	0.0275 (4)	
H3A	0.2050	0.2870	0.4944	0.033*	
H3B	0.4135	0.3150	0.4889	0.033*	
C4	0.3862 (3)	0.18769 (13)	0.54514 (8)	0.0292 (4)	
H4A	0.5117	0.1641	0.5416	0.035*	
H4B	0.3862	0.2251	0.5849	0.035*	
C5	0.2574 (2)	0.09592 (13)	0.54645 (8)	0.0259 (4)	
H5A	0.1351	0.1188	0.5551	0.031*	
H5B	0.3042	0.0502	0.5806	0.031*	
C6	0.2430 (2)	0.03854 (12)	0.48280 (7)	0.0211 (4)	
H6A	0.3608	0.0058	0.4780	0.025*	
H6B	0.1498	−0.0145	0.4835	0.025*	
C7	0.1869 (2)	0.05012 (11)	0.36345 (7)	0.0182 (3)	
C8	0.3726 (2)	−0.04708 (12)	0.29626 (8)	0.0251 (4)	
H8A	0.2760	−0.0984	0.2901	0.030*	
H8B	0.4911	−0.0822	0.3010	0.030*	
C9	0.3640 (2)	0.01985 (12)	0.23785 (8)	0.0221 (4)	
C10	0.3138 (2)	−0.02018 (13)	0.17760 (8)	0.0285 (4)	
H10	0.2810	−0.0886	0.1730	0.034*	
C11	0.3125 (2)	0.04149 (14)	0.12452 (8)	0.0293 (4)	
H11	0.2799	0.0162	0.0837	0.035*	
C12	0.3619 (2)	0.14262 (13)	0.13470 (8)	0.0254 (4)	
C13	0.4111 (2)	0.12222 (13)	0.24178 (8)	0.0247 (4)	
H13	0.4453	0.1496	0.2820	0.030*	
C14	0.0183 (2)	0.05485 (11)	0.33042 (7)	0.0185 (3)	
C15	−0.0991 (2)	0.11385 (12)	0.37036 (7)	0.0199 (4)	
C16	−0.0574 (2)	0.01791 (12)	0.26666 (7)	0.0190 (3)	
C17	−0.0880 (2)	−0.08572 (12)	0.25352 (8)	0.0233 (4)	
H17	−0.0618	−0.1332	0.2860	0.028*	
C18	−0.1565 (2)	−0.11919 (13)	0.19334 (8)	0.0263 (4)	
H18	−0.1736	−0.1885	0.1853	0.032*	
C19	−0.1993 (2)	−0.04852 (13)	0.14529 (8)	0.0253 (4)	
C20	−0.1746 (2)	0.05474 (12)	0.15634 (8)	0.0229 (4)	
H20	−0.2048	0.1020	0.1240	0.027*	
C21	−0.1040 (2)	0.08598 (11)	0.21652 (8)	0.0185 (3)	

### Atomic displacement parameters (Å^2^)

**Table:**

	*U*^11^	*U*^22^	*U*^33^	*U*^12^	*U*^13^	*U*^23^
Cl1	0.0450 (3)	0.0442 (3)	0.0269 (3)	−0.0021 (2)	−0.0017 (2)	−0.0110 (2)
Cl2	0.0338 (3)	0.0155 (2)	0.0269 (2)	0.00057 (16)	0.00361 (19)	0.00429 (15)
Cl3	0.0350 (3)	0.0439 (3)	0.0300 (3)	−0.0021 (2)	0.0051 (2)	0.00633 (19)
O1	0.0162 (6)	0.0236 (6)	0.0215 (6)	0.0040 (5)	0.0021 (5)	0.0022 (5)
O2	0.0166 (6)	0.0297 (6)	0.0280 (6)	0.0052 (5)	0.0021 (5)	0.0069 (5)
O3	0.0187 (6)	0.0257 (6)	0.0233 (6)	0.0067 (5)	0.0016 (5)	−0.0029 (5)
N1	0.0241 (8)	0.0266 (8)	0.0287 (8)	−0.0017 (6)	0.0034 (6)	−0.0031 (6)
C1	0.0152 (8)	0.0185 (8)	0.0226 (8)	0.0032 (6)	0.0020 (7)	0.0025 (6)
C2	0.0237 (9)	0.0211 (8)	0.0276 (9)	−0.0004 (7)	0.0062 (7)	0.0020 (7)
C3	0.0257 (10)	0.0208 (9)	0.0361 (10)	−0.0019 (7)	0.0043 (8)	−0.0039 (7)
C4	0.0313 (10)	0.0279 (9)	0.0277 (10)	−0.0004 (8)	−0.0006 (8)	−0.0068 (8)
C5	0.0262 (9)	0.0291 (9)	0.0221 (9)	0.0026 (7)	0.0002 (7)	0.0017 (7)
C6	0.0186 (8)	0.0201 (8)	0.0242 (9)	0.0013 (7)	−0.0005 (7)	0.0018 (7)
C7	0.0181 (8)	0.0148 (8)	0.0220 (8)	0.0015 (6)	0.0034 (7)	0.0037 (6)
C8	0.0233 (9)	0.0229 (9)	0.0292 (9)	0.0074 (7)	0.0027 (7)	−0.0058 (7)
C9	0.0156 (8)	0.0231 (9)	0.0279 (9)	0.0034 (7)	0.0038 (7)	−0.0053 (7)
C10	0.0291 (10)	0.0248 (9)	0.0326 (10)	−0.0035 (7)	0.0083 (8)	−0.0096 (8)
C11	0.0289 (10)	0.0354 (10)	0.0240 (9)	−0.0051 (8)	0.0050 (7)	−0.0105 (8)
C12	0.0168 (9)	0.0346 (10)	0.0255 (9)	−0.0004 (7)	0.0051 (7)	−0.0024 (7)
C13	0.0210 (9)	0.0291 (9)	0.0237 (9)	−0.0018 (7)	0.0009 (7)	−0.0071 (7)
C14	0.0182 (8)	0.0152 (8)	0.0221 (8)	0.0000 (6)	0.0020 (7)	0.0047 (6)
C15	0.0219 (9)	0.0170 (8)	0.0207 (8)	−0.0012 (7)	0.0019 (7)	0.0071 (6)
C16	0.0158 (8)	0.0186 (8)	0.0226 (8)	−0.0003 (6)	0.0022 (7)	0.0024 (6)
C17	0.0223 (9)	0.0184 (8)	0.0290 (9)	−0.0011 (7)	0.0019 (7)	0.0048 (7)
C18	0.0257 (10)	0.0199 (9)	0.0332 (10)	−0.0032 (7)	0.0031 (8)	−0.0023 (7)
C19	0.0220 (9)	0.0300 (9)	0.0237 (9)	−0.0023 (7)	0.0015 (7)	−0.0054 (7)
C20	0.0202 (9)	0.0267 (9)	0.0218 (8)	0.0022 (7)	0.0030 (7)	0.0039 (7)
C21	0.0175 (8)	0.0150 (8)	0.0234 (9)	0.0009 (6)	0.0041 (7)	−0.0003 (6)

### Geometric parameters (Å, °)

**Table:**

Cl1—C19	1.7398 (17)	C6—H6A	0.9700
Cl2—C21	1.7400 (16)	C6—H6B	0.9700
Cl3—C12	1.7440 (17)	C7—C14	1.345 (2)
O1—C15	1.366 (2)	C8—C9	1.507 (2)
O1—C1	1.4577 (18)	C8—H8A	0.9700
O2—C15	1.2090 (19)	C8—H8B	0.9700
O3—C7	1.3375 (18)	C9—C13	1.385 (2)
O3—C8	1.4421 (18)	C9—C10	1.387 (2)
N1—C12	1.320 (2)	C10—C11	1.378 (2)
N1—C13	1.342 (2)	C10—H10	0.9300
C1—C7	1.505 (2)	C11—C12	1.384 (2)
C1—C6	1.519 (2)	C11—H11	0.9300
C1—C2	1.534 (2)	C13—H13	0.9300
C2—C3	1.526 (2)	C14—C15	1.474 (2)
C2—H2A	0.9700	C14—C16	1.480 (2)
C2—H2B	0.9700	C16—C21	1.398 (2)
C3—C4	1.530 (2)	C16—C17	1.400 (2)
C3—H3A	0.9700	C17—C18	1.385 (2)
C3—H3B	0.9700	C17—H17	0.9300
C4—C5	1.524 (2)	C18—C19	1.384 (2)
C4—H4A	0.9700	C18—H18	0.9300
C4—H4B	0.9700	C19—C20	1.382 (2)
C5—C6	1.531 (2)	C20—C21	1.380 (2)
C5—H5A	0.9700	C20—H20	0.9300
C5—H5B	0.9700		
			
C15—O1—C1	109.69 (12)	C9—C8—H8A	108.9
C7—O3—C8	120.36 (13)	O3—C8—H8B	108.9
C12—N1—C13	116.43 (15)	C9—C8—H8B	108.9
O1—C1—C7	102.52 (12)	H8A—C8—H8B	107.7
O1—C1—C6	109.58 (12)	C13—C9—C10	117.49 (15)
C7—C1—C6	112.12 (13)	C13—C9—C8	121.71 (15)
O1—C1—C2	108.54 (12)	C10—C9—C8	120.77 (15)
C7—C1—C2	111.66 (12)	C11—C10—C9	119.95 (16)
C6—C1—C2	111.93 (14)	C11—C10—H10	120.0
C3—C2—C1	111.83 (13)	C9—C10—H10	120.0
C3—C2—H2A	109.2	C10—C11—C12	117.11 (16)
C1—C2—H2A	109.2	C10—C11—H11	121.4
C3—C2—H2B	109.2	C12—C11—H11	121.4
C1—C2—H2B	109.2	N1—C12—C11	125.13 (16)
H2A—C2—H2B	107.9	N1—C12—Cl3	115.93 (13)
C2—C3—C4	110.44 (14)	C11—C12—Cl3	118.94 (13)
C2—C3—H3A	109.6	N1—C13—C9	123.89 (15)
C4—C3—H3A	109.6	N1—C13—H13	118.1
C2—C3—H3B	109.6	C9—C13—H13	118.1
C4—C3—H3B	109.6	C7—C14—C15	105.99 (14)
H3A—C3—H3B	108.1	C7—C14—C16	133.52 (14)
C5—C4—C3	111.65 (14)	C15—C14—C16	120.44 (14)
C5—C4—H4A	109.3	O2—C15—O1	121.42 (14)
C3—C4—H4A	109.3	O2—C15—C14	128.58 (15)
C5—C4—H4B	109.3	O1—C15—C14	109.99 (13)
C3—C4—H4B	109.3	C21—C16—C17	116.66 (15)
H4A—C4—H4B	108.0	C21—C16—C14	121.05 (14)
C4—C5—C6	111.10 (14)	C17—C16—C14	122.28 (14)
C4—C5—H5A	109.4	C18—C17—C16	121.58 (15)
C6—C5—H5A	109.4	C18—C17—H17	119.2
C4—C5—H5B	109.4	C16—C17—H17	119.2
C6—C5—H5B	109.4	C19—C18—C17	119.35 (15)
H5A—C5—H5B	108.0	C19—C18—H18	120.3
C1—C6—C5	113.06 (13)	C17—C18—H18	120.3
C1—C6—H6A	109.0	C20—C19—C18	121.13 (16)
C5—C6—H6A	109.0	C20—C19—Cl1	119.24 (13)
C1—C6—H6B	109.0	C18—C19—Cl1	119.63 (13)
C5—C6—H6B	109.0	C21—C20—C19	118.36 (15)
H6A—C6—H6B	107.8	C21—C20—H20	120.8
O3—C7—C14	135.67 (15)	C19—C20—H20	120.8
O3—C7—C1	112.59 (13)	C20—C21—C16	122.90 (14)
C14—C7—C1	111.73 (13)	C20—C21—Cl2	117.74 (12)
O3—C8—C9	113.22 (12)	C16—C21—Cl2	119.32 (12)
O3—C8—H8A	108.9		
			
C15—O1—C1—C7	−2.79 (15)	C12—N1—C13—C9	−0.3 (2)
C15—O1—C1—C6	−122.03 (13)	C10—C9—C13—N1	0.2 (2)
C15—O1—C1—C2	115.46 (13)	C8—C9—C13—N1	178.20 (15)
O1—C1—C2—C3	68.12 (17)	O3—C7—C14—C15	179.93 (16)
C7—C1—C2—C3	−179.60 (13)	C1—C7—C14—C15	−1.50 (17)
C6—C1—C2—C3	−52.96 (18)	O3—C7—C14—C16	−2.6 (3)
C1—C2—C3—C4	55.70 (18)	C1—C7—C14—C16	175.99 (15)
C2—C3—C4—C5	−57.18 (19)	C1—O1—C15—O2	−179.05 (13)
C3—C4—C5—C6	55.15 (19)	C1—O1—C15—C14	2.09 (16)
O1—C1—C6—C5	−69.25 (17)	C7—C14—C15—O2	−179.10 (15)
C7—C1—C6—C5	177.61 (13)	C16—C14—C15—O2	3.0 (2)
C2—C1—C6—C5	51.22 (18)	C7—C14—C15—O1	−0.35 (16)
C4—C5—C6—C1	−52.32 (18)	C16—C14—C15—O1	−178.24 (13)
C8—O3—C7—C14	0.0 (3)	C7—C14—C16—C21	−109.1 (2)
C8—O3—C7—C1	−178.59 (12)	C15—C14—C16—C21	68.14 (19)
O1—C1—C7—O3	−178.43 (12)	C7—C14—C16—C17	71.7 (2)
C6—C1—C7—O3	−60.99 (17)	C15—C14—C16—C17	−111.08 (17)
C2—C1—C7—O3	65.55 (16)	C21—C16—C17—C18	1.7 (2)
O1—C1—C7—C14	2.66 (16)	C14—C16—C17—C18	−179.07 (15)
C6—C1—C7—C14	120.10 (15)	C16—C17—C18—C19	−1.3 (2)
C2—C1—C7—C14	−113.36 (15)	C17—C18—C19—C20	0.1 (3)
C7—O3—C8—C9	69.36 (18)	C17—C18—C19—Cl1	179.83 (12)
O3—C8—C9—C13	26.2 (2)	C18—C19—C20—C21	0.7 (2)
O3—C8—C9—C10	−155.88 (14)	Cl1—C19—C20—C21	−179.05 (12)
C13—C9—C10—C11	0.1 (2)	C19—C20—C21—C16	−0.3 (2)
C8—C9—C10—C11	−177.88 (16)	C19—C20—C21—Cl2	177.62 (12)
C9—C10—C11—C12	−0.3 (2)	C17—C16—C21—C20	−0.9 (2)
C13—N1—C12—C11	0.2 (2)	C14—C16—C21—C20	179.87 (14)
C13—N1—C12—Cl3	−178.92 (12)	C17—C16—C21—Cl2	−178.75 (12)
C10—C11—C12—N1	0.2 (3)	C14—C16—C21—Cl2	2.0 (2)
C10—C11—C12—Cl3	179.21 (13)		

### Hydrogen-bond geometry (Å, °)

**Table:**

*D*—H···*A*	*D*—H	H···*A*	*D*···*A*	*D*—H···*A*
C13—H13···O3	0.93	2.53	2.864 (2)	102

## References

[bb1] Cremer, D. & Pople, J. A. (1975). *J. Am. Chem. Soc* **97**, 1354–1358.

[bb2] Lu, Y., Tao, J. Z. & Zhang, Z. R. (2008). *Chem. Intermed.* **10**, 25–28.

[bb3] Rigaku (2005). *CrystalClear* Rigaku Corporation, Tokyo, Japan.

[bb4] Sarcevic, N., Zsindely, J. & Schmid, H. (1973). *Helv. Chim. Acta*, **56**, 1457–1476.

[bb5] Sheldrick, G. M. (2008). *Acta Cryst.* A**64**, 112–122. 10.1107/S0108767307043930 18156677

[bb6] Thomas, B., Jordi, B. B., Reiner, F. & Ralf, N. (2003). *Chimia*, **57**, 697–701.

