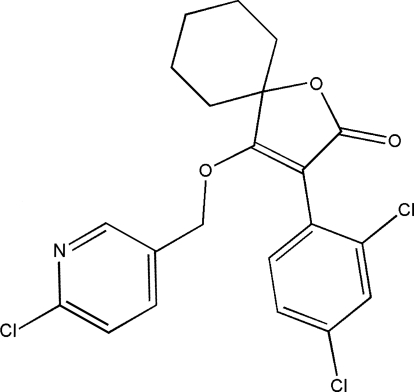# 4-[(6-Chloro-2-pyrid­yl)meth­oxy]-3-(2,4-dichloro­phen­yl)-1-oxaspiro­[4.5]dec-3-en-2-one. Corrigendum

**DOI:** 10.1107/S1600536809023290

**Published:** 2009-06-27

**Authors:** Liang-zhong Xu, Jin Huang, Qun-qun Su, Wei Guo

**Affiliations:** a College of Chemistry and Molecular Engineering, Qingdao University of Science and Technology, Qingdao 266042, People’s Republic of China

## Abstract

The chemical name of the title compound in the paper by Xu, Huang & Guo [*Acta Cryst.* (2009), E**65**, o846] is corrected and the structural diagram is updated.

## Abstract 2

Corrigendum to *Acta Cryst.* (2009), E**65**, o846.

In the paper by Xu, Huang & Guo [*Acta Cryst.* (2009), E**65**, o846], the chemical name given in the *Title* should be ‘4-[(6-Chloro-3-pyrid­yl)meth­oxy]-3-(2,4-dichloro­phen­yl)-1-oxa­spiro­[4.5]dec-3-en-2-one’. An updated structural diagram is shown below.